# 1α,25-Dihydroxyvitamin D3 Promotes Angiogenesis After Cerebral Ischemia Injury in Rats by Upregulating the TGF-β/Smad2/3 Signaling Pathway

**DOI:** 10.3389/fcvm.2022.769717

**Published:** 2022-03-16

**Authors:** Yajie Zhang, Yingfeng Mu, Hongmei Ding, Bo Du, Mingyue Zhou, Qingqing Li, Shitong Gong, Fuchi Zhang, Deqin Geng, Yanqiang Wang

**Affiliations:** ^1^Department of Neurology, Xuzhou Medical University, Xuzhou, China; ^2^Department of Neurology, The Affiliated Hospital of Xuzhou Medical University, Xuzhou, China; ^3^Department of Neurology, The Third Hospital of Huai'an, Huai'an, China; ^4^Department of Neurology II, The Affiliated Hospital of Weifang Medical University, Weifang, China

**Keywords:** 1α, 25-dihydroxyvitamin D3, vitamin D receptor, TGF-β/Smad2/3 signaling pathway, vascular endothelial growth factor, cerebral ischemia-reperfusion, angiogenesis

## Abstract

Stroke is a disease with high morbidity, disability and mortality, which seriously endangers the life span and quality of life of people worldwide. Angiogenesis and neuroprotection are the key to the functional recovery of penumbra function after acute cerebral infarction. In this study, we used the middle cerebral artery occlusion (MCAO) model to investigate the effects of 1α,25-dihydroxyvitamin D3 (1,25-D3) on transforming growth factor-β (TGF-β)/Smad2/3 signaling pathway. Cerebral infarct volume was measured by TTC staining. A laser speckle flow imaging system was used to measure cerebral blood flow (CBF) around the ischemic cortex of the infarction, followed by platelet endothelial cell adhesion molecule-1 (PECAM-1/CD31) and isolectin-B4 (IB4) immunofluorescence. The expression of vitamin D receptor (VDR), TGF-β, Smad2/3, p-Smad2, p-Smad3, and vascular endothelial growth factor (VEGF) was analyzed by western blot and RT-qPCR. Results showed that compared with the sham group, the cerebral infarction volume was significantly increased while the CBF was reduced remarkably in the MCAO group. 1,25-D3 reduced cerebral infarction volume, increased the recovery of CBF and expressions of VDR, TGF-β, p-Smad2, p-Smad3, and VEGF, significantly increased IB4^+^ tip cells and CD31^+^ vascular length in the peri-infarct area compared with the DMSO group. The VDR antagonist pyridoxal-5-phosphate (P5P) partially reversed the neuroprotective effects of 1,25-D3 described above. In summary, 1,25-D3 plays a neuroprotective role in stroke by activating VDR and promoting the activation of TGF-β, which in turn up-regulates the TGF-β/Smad2/3 signaling pathway, increases the release of VEGF and thus promotes angiogenesis, suggesting that this signaling pathway may be an effective target for ischemic stroke treatment. 1,25-D3 is considered to be a neuroprotective agent and is expected to be an effective drug for the treatment of ischemic stroke and related diseases.

## Introduction

For past two decades, thrombolytic therapy has been the standard treatment for acute ischemic stroke. Intravenous thrombolysis can be used to treat acute stroke only when it can be determined that the time after the onset of symptoms is less than 4.5 h (1, 2). Although its efficacy has been demonstrated in clinical trials, the number of patients benefited by this procedure is unfortunately low, around 5% of all stroke patients, a fact ascribed to the narrow time window for t-PA administration and because delayed thrombolytic therapy and blood reperfusion have been associated with a high risk of hemorrhagic transformation and oxidative stress, thus causing additional damage (3). A recent clinical study showed that in patients with acute stroke whose onset time was unknown, the incidence of intracranial hemorrhage was also significantly increased at 90 days after intravenous injection of alteplase under the guidance of diffusion-weighted imaging and FLAIR (fluid attenuated inversion recovery) mismatch in the ischemic area compared with the placebo group (4). Endovascular thrombectomy (EVT) has been shown to be a highly effective treatment in high-resource countries. Infrastructure and support are needed for EVT in the developing world (5). Therefore, it is particularly important to seek more therapeutic measures for ischemic stroke.

1α,25-dihydroxyvitamin D3 (1,25-D3) is widely expressed in human organs and tissues and exerts the steroid effect in the whole body. Its neuroprotective effect has been paid more and more attention by scholars. 1,25-D3 produces a wide range of biological activities once binding to vitamin D receptor (VDR), including inhibition of proliferation, affecting angiogenesis, regulating immune activity, and endocrine activity (6–8). Observational studies have shown that patients with lower serum 1,25-D3 levels experience infarct volume and worse functional outcomes after stroke, indicating that 1,25-D3 may play a protective role during cerebral ischemia, but whether it can promote brain function recovery in terms of angiogenesis has not been reported (9).

After the brain encounters ischemia and hypoxia, cells around the ischemic core suffer irreversible necrosis (10, 11). The injury around the infarction is selective and progressive, thus ensuring the survival of cells around the infarction and angiogenesis is an effective target for brain protection. Angiogenesis is the key for functional recovery of ischemic penumbra after acute cerebral infarction (12–14). Vascular endothelial growth factor (VEGF) is essential for angiogenesis and neovascularization, and the release of VEGF is regulated by neurotrophic factors including transforming growth factor β (TGF-β) (15). In recent years, more and more attention has been paid to the link between TGF-β signaling pathway and angiogenesis. TGF-β participates in angiogenesis by regulating the stability of capillaries (16). Maharaj et al. demonstrated that activated TGF-β acts on endothelial cells and pericytes to induce the production and the release of VEGF (17). VEGF and TGF-β are involved in the regulation of endothelial cell stability, ependymal cell function and periventricular permeability (17). Patients with scleroderma can lessen angiogenesis by inhibiting TGF-β signaling pathway (18). Nanda et al have confirmed that after TGF-β signal damage, angiogenesis decreases in non-atherosclerotic ischemic injury model, which weakens the ability of neovascularization to mature (19). A lot of evidence confirmed the crucial role of TGF-β signaling pathway in angiogenesis (20, 21).

We investigated the effects of 1,25-D3 on angiogenesis and explore the possible mechanism after ischemia. We found that 1,25-D3 protected against ischemic injury and improved stroke outcome 3 days after MCAO. 1,25-D3 promotes angiogenesis by activating VDR, up-regulating TGF-β/Smad2/3 signaling pathway and inducing the release of VEGF. Therefore, our study highlights the potential of 1,25-D3 in stroke treatment.

## Materials and Methods

### Ethics Statement

All animal experiments were conducted in accordance with the National Institute of Health Guide for the Care and Use of Laboratory Animals of Laboratory Animals. All the experimental procedures involved in this study were approved by the Animal Ethics Committee of Xuzhou Medical University (protocol: 202007A102).

### Animals

Male Sprague-Dawley (SD) rats were provided by the Experimental Animal Center of Xuzhou Medical University. All rats were kept in pathogen-free conditions and housed under a light-dark cycle for 12 h (20 ± 1°C, and 55 ± 10% humidity), with access to food and water *ad libitum*.

### Chemicals and Reagents

1,25-D3 and VDR antagonist pyridoxal-5-phosphate (P5P) were purchased from Med chem Express. 2,3,5-triphenyltetrazolium chloride (TTC) was purchased from Sigma-Aldrich. The primary antibodies: anti-VDR antibody (14526-1-AP) and anti-β-actin antibody (20536-1-AP) were purchased from Proteintech. Anti-VEGF antibody (ab1316) and anti-platelet endothelial cell adhesion molecule-1 (PECAM-1/CD31) antibody (ab222783) were purchased from Abcam. Anti- isolectin-B4 (IB4) antibody (DL-1207) was purchased from Vector Laboratories. The primary antibodies of anti-TGF-β (3711S), anti-Smad2/3 (8685T), anti-p-Smad2 (18338T) and anti-p-Smad3 (9250T) were purchased from Cell Signaling Technology. MCAO threads (2636-100, diameter of the head of nylon filament: 0.36 ± 0.02 mm) were purchased from Beijing Shadong Biotechnology. Reverse transcription kit, SYBRGreen^®^ PremixExTaq™ II kits were purchased from Takara.

### Establishment of the Middle Cerebral Artery Occlusion/Reperfusion (MCAO) and Grouping

A total of 120 male Sprague-Dawley rats weighing 250 to 280 g were used in this study. Rats were randomly assigned resulting in five groups, each consisting of 24 rats:sham group, middle cerebral artery ischemia-reperfusion group (MCAO), MCAO + dimethyl sulfoxide group (DMSO), MCAO + 1,25-D3 group (1,25-D3) and MCAO + 1,25-D3 + VDR antagonist (P5P, Pyridoxal-5-Phosphate) group (1,25-D3 + P5P). Rats from all the groups were subjected to a 90-min middle cerebral artery occlusion followed by reperfusion as previously described (10). The neurological function was scored according to the neurological deficit score established by Longa et al. (22). Grade 0: no neurological deficit; Grade I: mild focal neurological deficit (inability to fully extend the ischemic forelimb); Grade II: moderate focal neurological deficit (circling to the ischemic scar); Grade III: severe focal neurological deficit (dumping to the ischemic scar); Grade IV: lethargic and unable to walk spontaneously. Neurological severity scores of I to III were considered successful modeling, otherwise the corresponding number of rats was eliminated and supplemented. Rats of the 1,25-D3 group were administered intravenously with 1,25-D3 at a dose of 5 mg/kg (dissolved in 2% DMSO) 30 min before reperfusion, and the 1,25-D_3_ + P5P group was administered intravenously with 1,25-D3 at a dose of 5 mg/kg (dissolved in 2% DMSO) and P5P at a dose of 0.4 mg/kg (dissolved in 0.9% NaCl). The same volume of DMSO was given in the DMSO group intravenously. The drug was given once a day for 3 consecutive days.

### Cerebral Infarction Volume Assessment

After anesthesia, the brains were refrigerated at −20°C for 20 min and then consecutively sliced into coronal slices, each slice being about 2 mm thick. Then they were placed into 2%TTC and incubated at 37°C for 20 min. After staining, the brain slices were fixed overnight in 10% paraformaldehyde and photographed by camera. Image J was used to calculate the percentage of cerebral infarction volume. The percentage of cerebral infarct volume in rats (%) = (volume of the normal cerebral hemisphere - volume of the non-infarct cerebral hemisphere on the infarct side)/volume of the normal cerebral hemisphere ^*^100%.

### Cerebral Blood Flow Measurement

Cortical cerebral blood flow (CBF) was monitored by a laser speckle flow imaging technique 3 days after reperfusion. All procedures were performed under double-blind conditions. Briefly, rats were anesthetized and disinfected using iodophor, and the skull was exposed. The fascia attached to the skull was removed as much as possible and 0.9% saline was added to maintain the liquid level. Images and quantification of cerebral blood flow in the penumbra can be accessed by the laser speckle flow imaging technique (RFLSI III, RWD, China).

### Immunofluorescence

The rats in each group were sacrificed 3 days after reperfusion and fixed for immunofluorescence of CD31 and IB4. Sections were incubated with 5% BSA for 1 h and then overnight with anti-CD31 antibody (1:200) at 4°C. After washing with PBST (0.1% Triton X-100 in 0.1 M PBS), fluorescent secondary antibody (1:500) and IB4 were incubated at room temperature for 1 h, then incubated with DAPI. Ultra-high resolution inverted fluorescence microscope (Stellaris5, Leica) was used for observation, and appropriate fluorescent light source was selected, and photos were taken. The vascular length and tip cell count was quantified by Image J.

### Western Blot Analysis

The fresh tissue around the cerebral infarction was cryopreserved with liquid nitrogen, homogenized, centrifuged, and the total protein was extracted. The protein concentration was detected by BCA protein assay kit (Beyotime Biotechnology) and the 5 ug/ul system was prepared. Protein was boiled at 100°C for 5 min, then separated by SDS-PAGE and transferred to NC membrane (Cytiva). After incubated with 5% skimmed milk at room temperature for 2 h, incubated with the primary antibodies at 4°C overnight (1:1,000), incubated with the secondary antibodies at room temperature for 1 h (1: 10,000), and developed by ECL colorimetric method. The Image J was used to measure the gray value of the strip. The expression of the target protein band is expressed by the gray value of the target protein band/β-actin gray value.

### Quantitative Real-Time PCR

Total RNA was extracted from peri-infarct tissues by Trizol. Complementary deoxyribonucleic acid (cDNA) was reverse transcribed and used as a template for the reaction by fluorescence quantitative real-time PCR apparatus. The target gene fragment was amplified according to the steps of the Power SYBR Green PCR Master Mix kit (Takara). Real-time PCR reaction conditions were as follows: pre-denaturation at 95°C for 10 s, denaturation at 95°C for 5 s, annealing and extension at 60°C for 20 s, a total of 40 cycles. After the reaction was complete, the dissolution curve program was run to detect the specificity of the amplified products. The following primers were used: TGF-β1_*fwd* 5'-3': TGAGTGGCTGTCTTTTGACG, TGF-β1_*rev* 5'-3': GGTTCATGTCATGGATGGTG. TGF-β2_*fwd* 5'-3': GTGATTTCCATCTACAACAGTACC, TGF-β2_*rev* 5'-3': TATAAACCTCCTTGGCGTAGTAC; TGF-β3_*fwd* 5'-3': CCCAACCCCAGCTCCAAGCG; TGF-β3_*rev* 5'-3': AGCCACTCGCGCACAGTGTC; VDR_*fwd* 5'-3': CCACCGGCAGAAACGTGTAT; VDR_rev 5'-3': TGCCTTGTGAGAGGCTCTAGGA; VEGF_*fwd* 5'-3': CCGTCCTGTGTGCCCCTAATG; VEGF_*rev* 5'-3': CGCATGATCTGCATAGTGACGTTG; GAPDH_*fwd* 5'-3': GCATCTTCTTGTGCAGTGCC and GAPDH_*rev* 5'-3': TACGGCCAAAT CCGTTCACA.

### Statistical Analysis

GraphPad Prism 5 (Graph Pad Software Inc, La Jolla, CA) was used to generate graphs and perform statistical analysis. Normal distribution was determined by the Kolmogorov-Smirnov test. One-way ANOVA was used for comparison among multiple groups, and Turkey′s *post hoc* test was used for further pairwise comparison. Data were expressed as Mean ± SEM. Differences were considered statistically significant at *p* < 0.05.

## Results

### 1,25-D3 Significantly Reduced the Volume of Infarct Regions, Alleviated Brain Injury in Rats With Stroke

**Figure 1:**
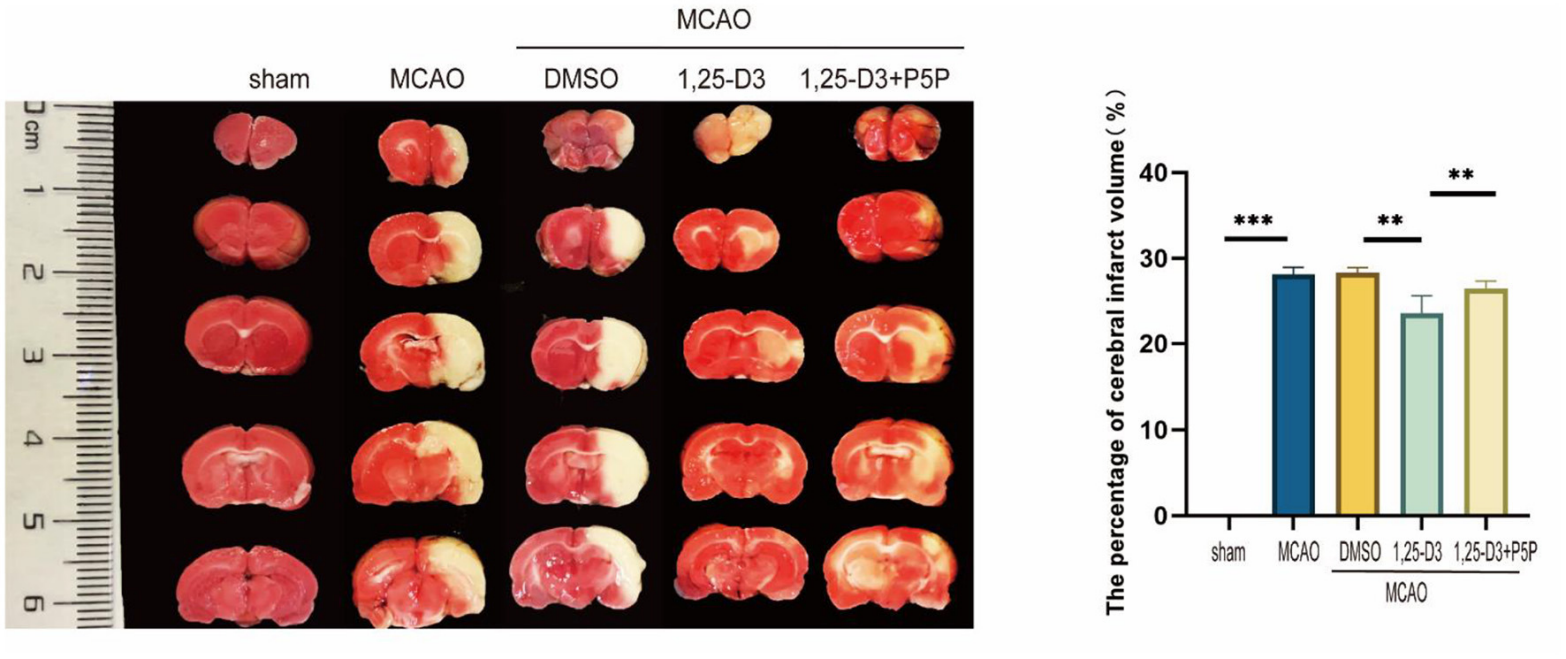
1,25-D3 reduced the volume of infarct regions in rats 3 days after stroke. Data were represented as Mean ± SEM, *n* = 5 per group, *p < 0.05, **p < 0.005, ***p < 0.001. MCAO, middle cerebral artery occlusion; DMSO, dimethyl sulfoxide; P5P, antagonist of VDR; 1,25-D3, 1α,25-dihydroxyvitamin D3.

In order to confirm the effect of 1,25-D3 on stroke, we analyzed the volume of cerebral infarction 3 days after MCAO. Compared with sham group, the volume of cerebral infarction in MCAO group was significantly increased. There was no significant difference in cerebral infarction volume in DMSO and MCAO group. The cerebral infarct volume was decreased in 1,25-D3 group compared with the DMSO group. While it was more in 1,25-D3 + P5P group than 1,25-D3 group (Figure 1). It is suggested that 1,25-D3 treatment can significantly reduce the cerebral infarct volume and reduce the brain injury in stroke rats. VDR antagonist P5P partially reversed the neuroprotective effect of 1,25-D3.

### 1,25-D3 Promoted the Recovery of CBF After Ischemia-Reperfusion

**Figure 2:**
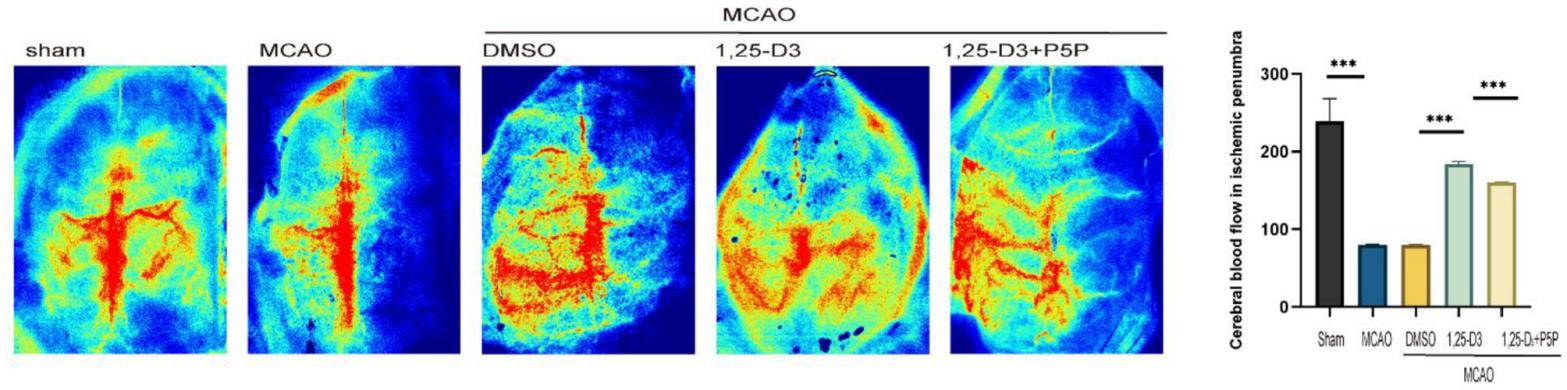
1,25-D3 increased cerebral blood flow of peri-infarcted cortex 3 days after stroke, and P5P partially reversed the neuroprotective effect. Data were represented as Mean ± SEM, *n* = 5 per group, *p < 0.05, **p < 0.005, ***p < 0.001. MCAO, middle cerebral artery occlusion; DMSO, dimethyl sulfoxide; P5P, antagonist of VDR; 1,25-D3, 1α,25-dihydroxyvitamin D3.

We analyzed the CBF of peri-infarcted cortex in different groups of rats 3 days after ischemia-reperfusion using a laser speckle imaging system. The results showed that compared with the sham group, the CBF around the infarct cortex was significantly decreased in the MCAO group. Compared with the DMSO group, the CBF at the infarct margin was increased in the 1,25-D3 group, which was decreased in the 1,25-D3 + P5P group (Figure 2). Namely, 1,25-D3 increased CBF recovery in ischemia-reperfusion rats, while VDR antagonist P5P partially reversed the neuroprotective effect of 1,25-D3.

### 1,25-D3 Increased Neovascularization and Improves Vascular Function After Stroke

**Figure 3:**
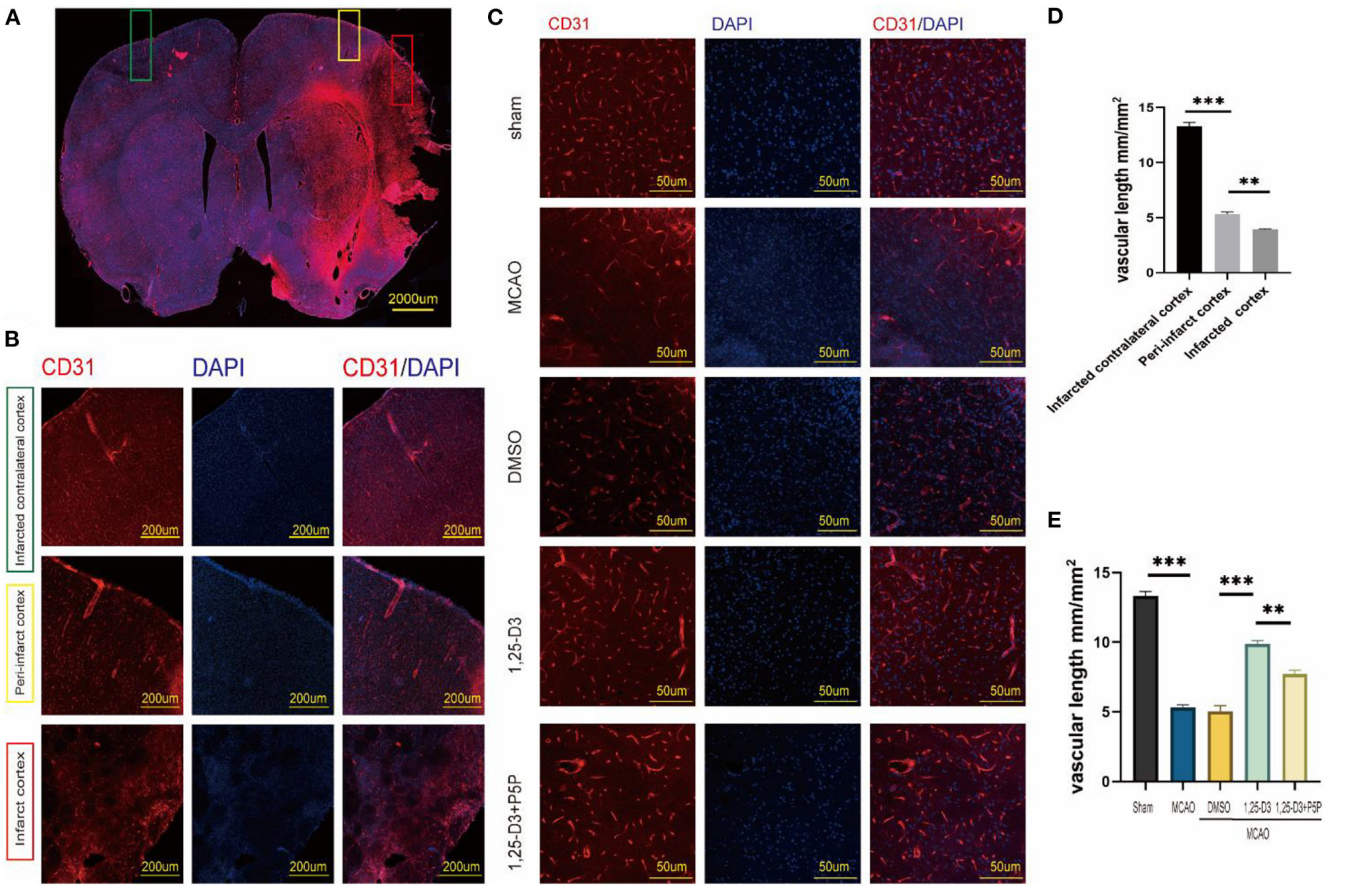
1,25-D3 increased the microvascular length and density and alleviated impairments induced by cerebral ischemic reperfusion 3 days after MCAO. **(A,B,D)** *In situ* expression of CD31 (red) and 4',6-diamidino-2-phenylindole (DAPI; blue) in different region of MCAO group (100× magnification 100). **(C,E)** *In situ* expression of CD31 (red) and 4',6-diamidino-2-phenylindole (DAPI; blue) in the peri-infarct region of sham, MCAO, DMSO, 1,25-D3 and 1,25-D3 + P5P groups (400× magnification 400). Data were represented as Mean ± SEM, *n* =5 per group. *p < 0.05, **p < 0.005, ***p < 0.001. MCAO, middle cerebral artery occlusion; 1,25-D3, 1α,25-dihydroxyvitamin D3.

The results of CD31 staining suggested that the length of vessel at various positions after ischemia-reperfusion differ. The microvascular length was longer than that in the infarct core area (Figures 3A,B,D). Comparison of different groups showed that the microvascular length was lower in the MCAO group than in the sham group, higher in the 1,25-D3 group than in the DMSO group. In addition, the microvascular length in 1,25-D3+P5P group was lower than in the 1,25-D3 group, suggesting that P5P can partially reduce the microvascular increase induced by 1,25-D3 (Figures 3C,E).

**Figure 4:**
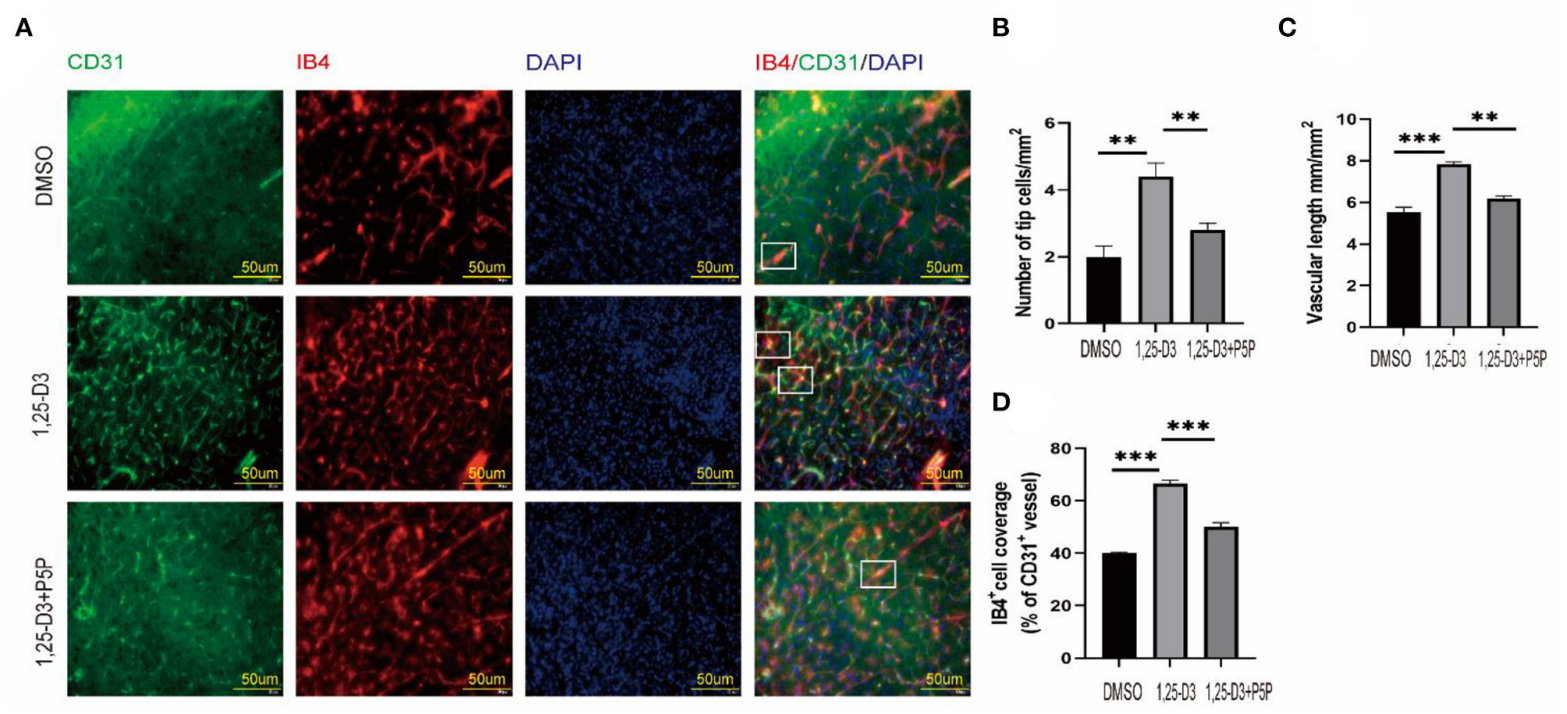
1,25-D3 increased neovascularization and improved vascular function 3 days after stroke. **(A)** *In situ* expression of CD31 (green), IB4 (red) and 4',6-diamidino-2-phenylindole (DAPI; blue) in the peri-infarct region of DMSO, 1,25-D3 and 1,25-D3 + P5P groups (400× magnification 400). Levels of IB4^+^ tip cells **(B)**, CD31^+^vascular length (mm/mm^2^ of brain tissue) **(C)**, and IB4^+^cell coverage (area ratio of IB4^+^ and CD31^+^) **(D)** in the peri-infarct region of DMSO, 1,25-D3 and 1,25-D3 + P5P groups. Data were represented as Mean ± SEM. Data were represented as Mean ± SEM, *n* = 5 per group, *p < 0.05, **p < 0.005, ***p < 0.001. MCAO, middle cerebral artery occlusion; DMSO, dimethyl sulfoxide; P5P, antagonist of VDR; 1,25-D3, 1α,25-dihydroxyvitamin D3.

To further understand the role of 1,25-D3 in neovascular development, we used IB4 fluorescence staining to look for neovascular sprouting as it is a marker of angiogenesis after we have confirmed that 1,25-D3 could increase CD31-labeled microvascular density after ischemia-reperfusion. IB4 staining showed that 1,25-D3 increased IB4^+^ tip cells in the peri-infarct area while P5P reversed the improvement of vascular development induced by 1,25-D3 (Figure 4).

### 1,25-D3 Promotes the Expression of VDR, VEGF, and TGF-β/Smad2/3 Signal Pathway Proteins in MCAO Rats

**Figure 5:**
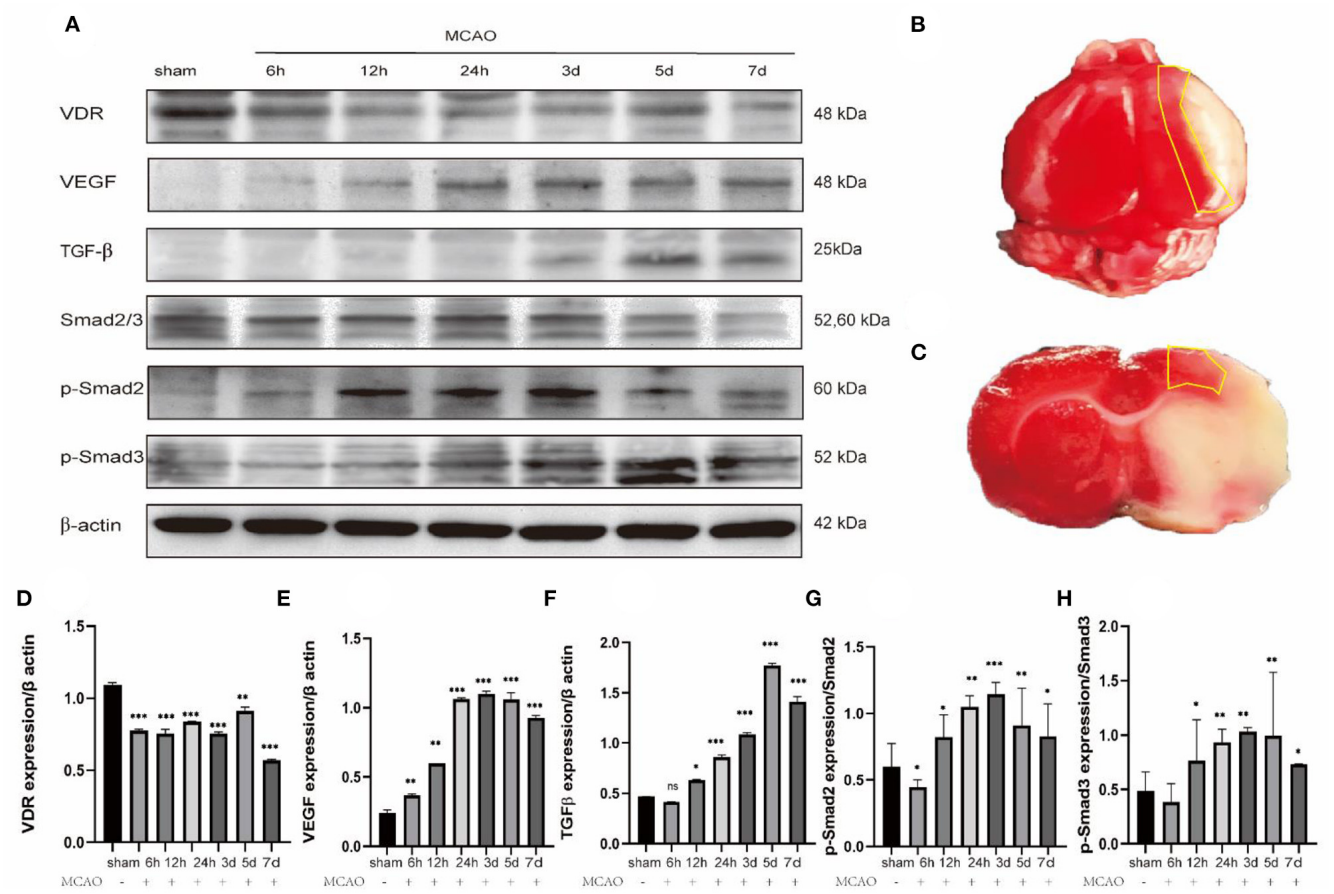
Changes in the expression of relevant proteins at different time after ischemia reperfusion. **(A,D–H)** Western blots shows the expression levels of VDR, VEGF, TGF-β, Smad2/3, p-Smad2, p-Smad3 differ at different time after ischemia reperfusion in the ischemic penumbra cortex. Data were represented as Mean ± SEM, *n* =3 per group. *p < 0.05, **p < 0.005, ***p < 0.001. **(B,C)** The yellow box selection indicates the area of the ischemic penumbra.

**Figure 6:**
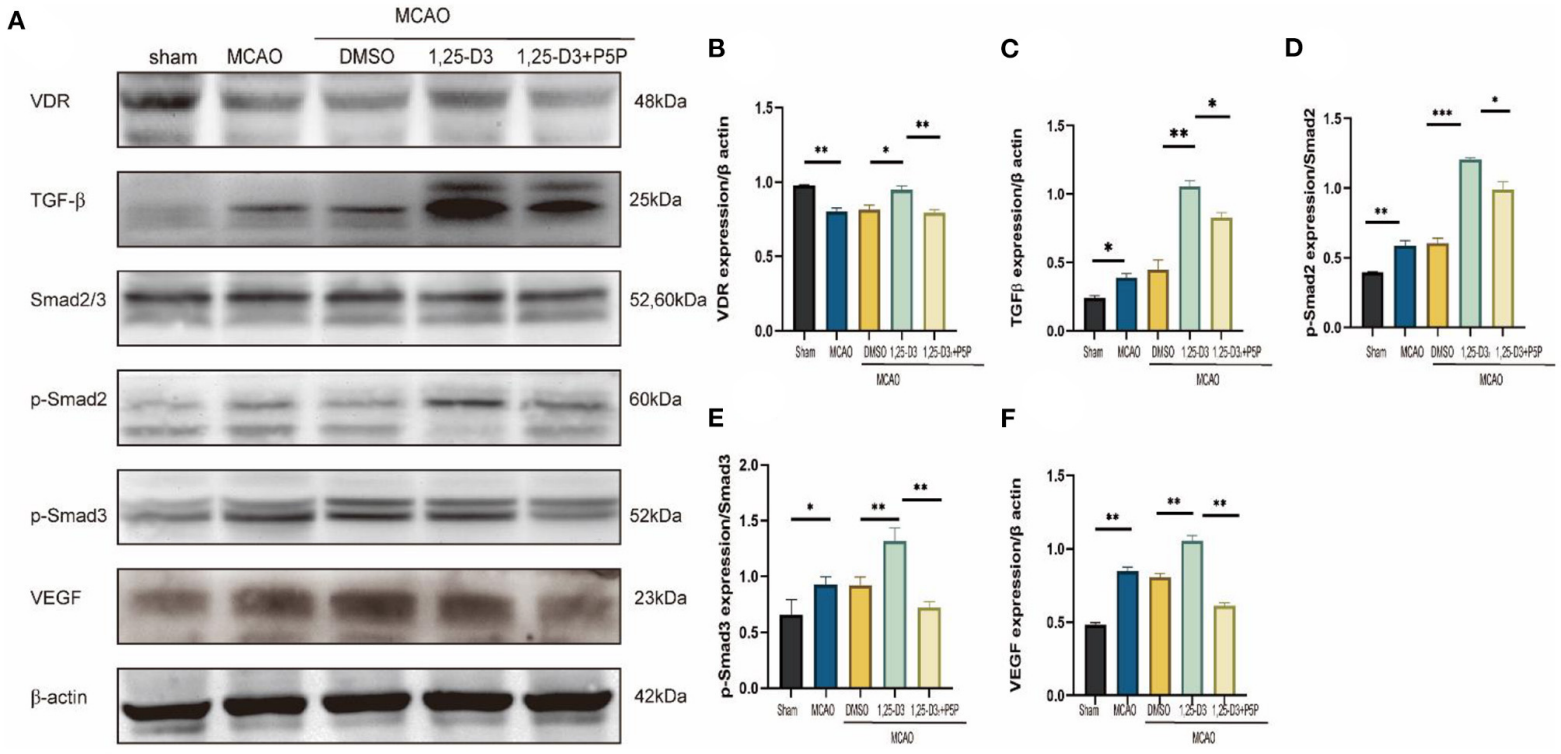
1,25-D3 promoted the expression of VDR, TGF-β, p-Smad2, p-Smad3, and VEGF induced by cerebral ischemic reperfusion 3 days after MCAO. **(A)** Western blot showed the expression of VDR, TGF-β, Smad2/3, p-Smad2, p-Smad3, VEGF in the ischemic penumbra cortex. Quantification of VDR **(B)**, TGF-β **(C)**, p-Smad2 **(D)**, p-Smad3 **(E)**, VEGF **(F)** in **(A)**. Data were represented as Mean ± SEM, *n* = 3 per group, *p < 0.05, **p < 0.005, ***p < 0.001. MCAO, middle cerebral artery occlusion; DMSO, dimethyl sulfoxide; P5P, antagonist of VDR; 1,25-D3, 1α,25-dihydroxyvitamin D3.

In order to explore the angiogenic effects of 1,25-D3 on TGF-β/Smad2/3 signaling pathway, we analyzed the expression of VEGF, TGF-β/Smad2/3 and VDR at different time points: 6 h, 12 h, 24 h, 3 d, 5 d, 7 d after reperfusion. We found that compared with the sham group, the expression of VDR decreased gradually at 6 h after ischemia-reperfusion. VEGF increased gradually at 12 h after stroke, reaching the peak at 3 d and then decreased slowly. The expression of TGF-β increased gradually at 12 h after stroke and reached the peak at 5 d. The phosphorylation levels of Smad2 and Smad3 increased at 12 h after reperfusion, reached the peak at day 3 after reperfusion, and then decreased gradually (Figure 5). We chose 3 days after reperfusion as the time point to measure the expression of angiogenesis-related proteins induced by 1,25-D3. Results showed that the expression of VDR, TGF-β, p-Smad2/Smad2, p-Smad3/Smad3, and VEGF in the periinfarcted cortex was significantly higher than DMSO group 3 days after stroke. The effect can be partially reversed by P5P. These results were confirmed by western blotting (Figure 6).

### The Expression of VDR, VEGF, and TGF-β1, TGF-β2, TGF-β3 mRNA in MCAO Rats Was Increased After Pretreatment With 1,25-D3

**Figure 7:**
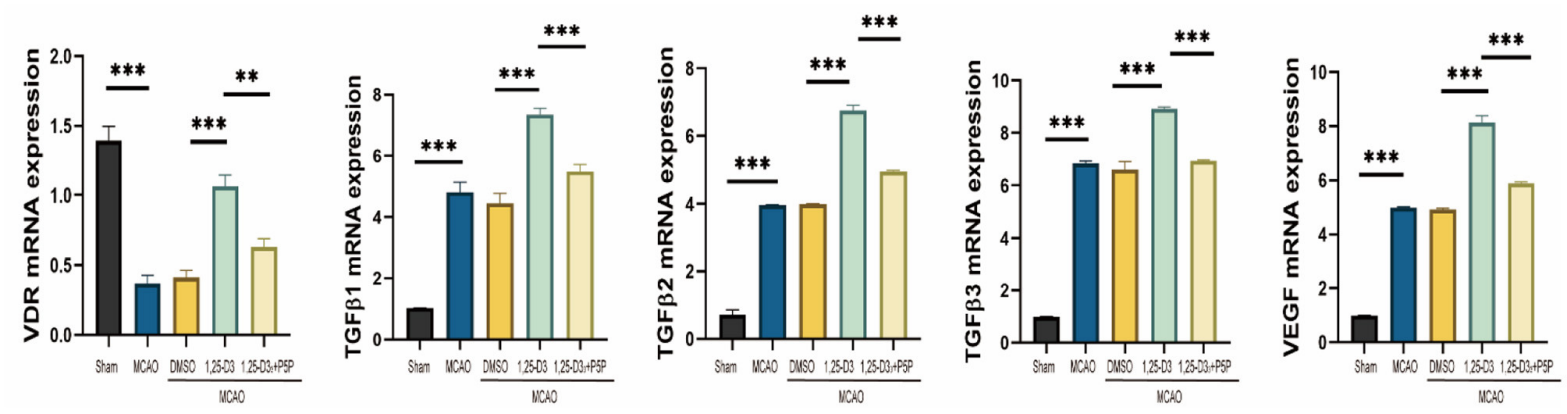
The expression of VDR, VEGF and TGF-β1, TGF-β2, TGF-β3 mRNA in MCAO rats increased after pretreatment with 1,25-D3. Data were represented as Mean ± SEM, *n* = 5 per group, *p < 0.05, **p < 0.005, ***p < 0.001. MCAO, middle cerebral artery occlusion; DMSO, dimethyl sulfoxide; P5P, antagonist of VDR; 1,25-D3, 1α,25-dihydroxyvitamin D3.

RT-qPCR analysis reveals the expression of VDR, TGF-β1, TGF-β2, TGF-β3, and VEGF mRNA levels in the peri-infarct cortex after 1,25-D3 treatment. The results showed that compared with the sham group, the expression of VDR mRNA decreased in MCAO rats. 1,25-D3 activated the VDR, TGF-β1, TGF-β2, TGF-β3 and VEGF mRNA level induced by ischemia, while the increase was partially reversed by P5P treatment (Figure 7). Therefore, 1,25-D3 promotes angiogenesis after stroke by activating VDR, thus promotes the activation of TGF-β subtypes and the expression of VEGF mRNA.

## Discussion

The strength and novelty of present study was that we demonstrated a salutary effect of 1,25-D3 on angiogenesis after ischemia stroke. 1,25-D3 activated VDR then up-regulated TGF-β/Smad2/3 signaling pathway and enhanced VEGF production, which contribute to promoting angiogenesis and improving stroke outcomes in rats after stroke.

We confirmed that 1,25-D3 reduces infarct volume and has a neuroprotective effect after cerebral ischemia injury in rats by TTC staining. Since our group previously demonstrated that 1,25-D3 reduces infarct size and attenuates neuronal cell death via PPAR-γ during cerebral ischemia (10). What is more, we find that 1,25-D3 can increase the CBF. As shown by CD31 and IB4 staining, micro-vascular density and length in the peri-infarct cortex was significantly higher in the 1,25-D3 group than in the DMSO and 1,25-D3 + P5P group, which has accompanied by increase in the number of new vessel sprouts, thereby playing a role in promoting angiogenesis. Compensatory angiogenesis and new capillaries improve blood perfusion around the ischemic area, providing a suitable microenvironment for nerve cell repair and promoting the recovery of neurological function after ischemic stroke (23). However, compensatory angiogenesis is often insufficient (24, 25). In order to explore the possible mechanism of promoting angiogenesis, we analyzed the expression of VDR, VEGF, TGF-β, and Smad2/3 at different time points after ischemia-reperfusion. At the end, we found that the expression of the corresponding proteins differs at different time points after ischemia-reperfusion. In western blot and RT-qPCR, we were surprised to find that 1,25-D3 could promote the expression of VDR, VEGF and TGF-β signaling pathway proteins 3 days after infarction. Because of this, we speculate that the proangiogenic effect of 1,25-D3 may be related to the TGF-β/Smad2/3 signaling pathway. Previous studies have shown that 1,25-D3 can promote the activation of PPAR-γ, while PPAR-γ and TGF-β are involved in the regulation of angiogenesis in the central nervous system (26, 27). Our results showed that reduction of VDR expression was strongly up-regulated by 1,25-D3 in rats under the condition of ischemia-reperfusion. VDR affects downstream proteins by inducing TGF-β/Smad2/3 signaling pathway. In response to 1,25-D3, the TGF-β/Smad2/3 signaling pathway is enhanced and acts as its activator to regulate downstream signaling (28–30). A large body of literature suggests that TGF-β, its receptors, and mediators of its downstream signaling are attractive targets for therapeutic interventions (20, 31, 32). TGF-β exerts its effects on effects mainly by upregulating the expression of proteins through TGF-β/Smad2/3 signaling (30, 33–35).

Lack of 1,25-D3 is a decisive causative factor in several neurodegenerative and neuropsychiatric disorders (36, 37). Once 1,25-D3 linked to the VDR, abundant biological effects can be exerted (8, 38). Mounting evidences have shown that 1,25-D3 plays a critical role in the process of stroke, including regulating the expression of neurotrophic factors and hormonal (36, 39, 40). It is involved in immune cell differentiation, gut microbiota modulation, gene transcription, blood-brain barrier integrity and so on (36, 39, 41). Further studies are needed to validate TGF-β isoforms, and their receptor subtypes involved in the TGF-β/Smad2/3 pathway.

In conclusion, we have demonstrated that 1,25-D3 promotes angiogenesis of the cortex around the ischemic boundary zone. Our results suggest that 1,25-D3 promotes angiogenesis by up-regulating TGF-β/Smad2/3 signaling pathway via VDR activation, thereby alleviating ischemia/reperfusion injury and improving stroke outcomes in rats.

Our study has several limitations to be resolved in the future. In this study, we show the effect of 1,25-D3 act on TGF-β/Smad2/3 in stroke. While TGF-β has several subtypes as TGF-β1 TGF-β2, TGF-β3 and so on. Future work should extend to the mechanism study for detail. In addition, no VDR overexpression group was set up in this study, and the effect of VDR was verified only by the antagonist group, thus plasmid construction will be performed in the next assay. Moreover, few studies address the effect of 1,25-D3 response on long-term outcome during late stage of stroke. Therefore, future studies are required to determine the potential impact of 1,25-D3 during stroke recovery.

## Data Availability Statement

The original contributions presented in the study are included in the article/Supplementary Material, further inquiries can be directed to the corresponding author/s.

## Ethics Statement

The animal study was reviewed and approved by the Animal Ethics Committee of Xuzhou Medical University (protocol: 202007A102).

## Author Contributions

Main manuscript text was written by YZ and YM. YZ, YM, HD, BD, MZ, QL, SG, and FZ performed the experiments and data analysis. DG and YW designed the study and revised the manuscript. All authors approved the final version of this manuscript.

## Funding

This work was supported by the National Natural Science Foundation of China (No. 81870943) and the Shandong Provincial Nature Fund Joint Special Fund Project (ZR2018LH006).

## Conflict of Interest

The authors declare that the research was conducted in the absence of any commercial or financial relationships that could be construed as a potential conflict of interest.

## Publisher's Note

All claims expressed in this article are solely those of the authors and do not necessarily represent those of their affiliated organizations, or those of the publisher, the editors and the reviewers. Any product that may be evaluated in this article, or claim that may be made by its manufacturer, is not guaranteed or endorsed by the publisher.
